# Participatory approaches and open data on venomous snakes: A neglected opportunity in the global snakebite crisis?

**DOI:** 10.1371/journal.pntd.0006162

**Published:** 2018-03-08

**Authors:** Lester Darryl Geneviève, Nicolas Ray, François Chappuis, Gabriel Alcoba, Maria Rosa Mondardini, Isabelle Bolon, Rafael Ruiz de Castañeda

**Affiliations:** 1 Institute of Global Health, Faculty of Medicine, University of Geneva, Geneva, Switzerland; 2 EnviroSPACE Lab, Institute for Environmental Sciences, University of Geneva, Geneva, Switzerland; 3 Division of Tropical and Humanitarian Medicine, University Hospitals of Geneva, Geneva, Switzerland; 4 Médecins Sans Frontières, Geneva, Switzerland; 5 Citizen Cyberlab, CERN-UNITAR-University of Geneva, Geneva, Switzerland; University of Melbourne, AUSTRALIA

## Snakebite: A humanitarian and data crisis

With over 100,000 human deaths and 400,000 cases of physical disability globally every year [1], snakebite is a major neglected tropical killer dramatically affecting poor and rural communities in Africa, Latin America, and Asia [2]. These communities have no political voice, and Médecins Sans Frontières refers to a “public health emergency gone under the radar,” stressing the lack of reliable reporting systems and an important underestimation of the global snakebite burden [1, 2, 3]. In addition, pharmaceutical companies have poor market incentive for antivenom production [2], and the recognition of snakebite at the World Health Organization (WHO) has fluctuated over recent years, lacking sufficient political and scientific support and only making it back on to the list of Neglected Tropical Diseases (NTDs) in the World Health Assembly (WHA) of 2017 [4].

In 2010, WHO identified 219 medically important venomous snakes (MIVS) and mapped their global distribution based on scientific literature, published reference texts, museum collection databases, and expert opinion [1]. These maps help identify at-risk populations to provide lifesaving care where needed [1]. However, snake ecology is complex, and our understanding of the geographical distribution of MIVS is limited [2, 5], especially considering current global anthropogenic changes (e.g., climate change and urbanization) [5]. The ecology of zoonoses such as avian influenza and their spread through animal movements have been well studied [6]. Besides the work of professionals, new approaches based on citizen participatory surveillance (e.g., birdwatchers) and mobile technologies have considerably improved the amount of data collected on animal distribution and our understanding of disease ecology [7]. So-called “citizen science” (CS) [7, 8] and “action ecology” [9] could be applied to other animals relevant to public health, including snakes. To our knowledge, the 2010 WHO database did not receive any contribution from CS projects. Similarly, open-access biodiversity databases such as the Global Biodiversity Information Facility (GBIF) (www.gbif.org) and VertNet (www.vertnet.org) have promoted biological research in recent years [9, 10] but remain poorly exploited in public health and applied herpetology. These two platforms contain both historical and recent data resulting from cooperation between institutions, enhancing data retrieval and sharing [9]. The GBIF platform also gathers data from CS projects, such as those from the Cornell Lab of Ornithology.

This study aims to build the first global observational map of MIVS based on crowdsourcing existing snake observations from a CS project and these two open-access biodiversity platforms, GBIF and VertNet. With over 715 million and 50 million observations respectively, these are massive platforms that gather global information on animals [10]. The study also aims to identify data gaps in currently available online data for MIVS distribution but does not intend to add new data on snakebite. More widely, the goal of this study is to highlight interest in and discuss the limitations of participatory approaches and open data in the context of MIVS ecology and their application for more specific public health questions around snakebite.

## Open global datasets on MIVS

We created a project titled “Medically Important Venomous Snakes” on February 4, 2017, on iNaturalist (www.inaturalist.org), a major CS platform dedicated to biodiversity [11]. We gathered existing geolocalized MIVS observations from iNaturalist user accounts from February 4 to February 15, 2017. Only “research-grade” observations were considered to ensure quality of identifications. These are defined by iNaturalist as observations having the agreement of more than two-thirds of identifiers in the user community on their taxonomic identification. Additional geolocalized observations were collected from five CS projects available on GBIF. After removal of redundant observations, all observations were merged into a single dataset (citizen-generated observations). These are generated by a potentially heterogeneous community of volunteer citizens with or without scientific background and with differing motivations. A second dataset consisting of traditional scientific sources of geolocalized MIVS observations (scientist-generated observations) was built from GBIF and VertNet on February 26, 2017. These observations are generated by scientists in the field as part of their research projects. All observations from these three sources of data (iNaturalist, GBIF, and VertNet) were identified at the species level.

## Data analysis

A total of 9,113 citizen-generated and 70,697 scientist-generated observations were statistically and spatially analysed using STATA 14 and QGIS 2.18.2. Descriptive statistics for Global Burden of Disease (GBD) regions were obtained for both types of observations. Estimates of envenoming and mortality were obtained from Kasturiratne et al. (2008) [12] to calculate snakebite-induced mortality rate per GBD region, and a linear regression analysis was performed between the log-transformed mortality rate and the number of observations. To infer the spatial congruence between both types of observations, we performed a linear regression analysis between the log-transformed numbers of citizen-generated and scientist-generated observations in the United States of America for the period 1990–2017. Moreover, distribution patterns of the four most frequently observed MIVS (*Agkistrodon contortrix*, *A*. *piscivorus*, *Crotalus atrox*, and *C*. *oreganus*) in our citizen-generated observations were compared to their respective species range maps for the US. The time frame of these observations was 1990–2017, and the species range maps were provided by the International Union for Conservation of Nature (IUCN). A points-in-polygon analysis was carried out for each species to determine the percentage of observations falling within range. A detailed version of the methodology is provided as supporting information (S1 Text).

## Results

**Fig 1:**
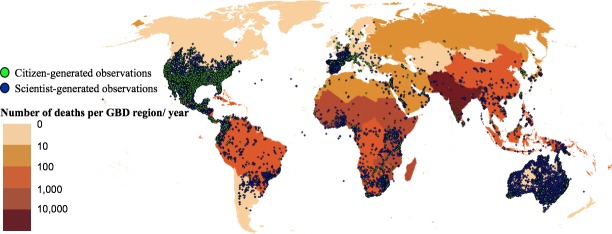
Geographical distribution of MIVS citizen-generated (*n* = 9,113) and scientist-generated (*n* = 70,697) observations on global map illustrating snakebite mortality for GBD regions of the world. The time frames for citizen-generated and scientist-generated observations are from 1967 to 2017 and 1700 to 2016 respectively. The color scale illustrates the mortality for these regions based on figures provided by Kasturiratne et al. (2008) [12]. Source: Made with Natural Earth. Free vector and raster map data available from www.naturalearthdata.com. The map (countries cultural theme version 3.1.0) is adapted and projected in the World Robinson coordinate reference system on QGIS 2.18.2. **Abbreviations:** GBD, global burden of disease; MIVS, medically important venomous snakes.

Citizen-generated and scientist-generated observations included respectively 55.7% and 80.8% of species listed by WHO as MIVS and covered 96 and 137 countries from all GBD regions of the world (Table 1). However, the global distribution of observations was strongly biased, with high-income North America, particularly the US, concentrating more than 79% (*n* = 7,207) of all citizen-generated observations and more than 34% (*n* = 24,134) of all scientist-generated observations (Fig 1 and Table 2). High-income North America had the highest overlap between citizen-generated and scientist-generated observations, and both groups coincided in 64% of all the species observed for this region (Table 2). The log-transformed number of citizen-generated observations was positively correlated with the log-transformed number of scientist-generated observations across the US (*p* < 0.01, r = 0.177–0.571, *n* = 79–466). The most frequently observed species and genus coincided between the two types of observations in 10 GBD regions (Table 3).

**Table 1:** General characteristics of citizen-generated observations and scientist-generated observations datasets.

Characteristics	Datasets	
	Citizen-generated observations	Scientist-generated observations
Time frame of observations	1967–2017	1700–2016
Number of observations	9,113	70,697^a^
Number of countries with observations	96	137
Number of observed species from WHO MIVS list (219 species)	122 (55.7%)	177 (80.8%)

^a^ Among the 70,697 scientist-generated observations, 20,049 did not have their date of observation.

**Abbreviations:** MIVS, medically important venomous snakes; WHO, World Health Organization.

**Table 2:** Geographical distribution of citizen-generated and scientist-generated snake species observations.

GBD regions	Citizen-generated observations		Scientist-generated observations		Species coincidence (%)^a^
	No. of observations *n* = 9,113	No. of MIVS species	No. of observations *n* = 70,697	No. of MIVS species	
Andean Latin America	44 (0.5%)	9	606 (0.9%)	18	50
Australasia	85 (0.9%)	9	17,920 (25.3%)	15	60
Caribbean	8 (0.1%)	2	200 (0.3%)	13	15
Central Asia	25 (0.3%)	6	50 (0.1%)	7	44
Central Europe	24 (0.3%)	3	32 (0.0%)	3	100
Central Latin America	693 (7.6%)	32	9,434 (13.3%)	47	65
Central sub-Saharan Africa	10 (0.1%)	7	422 (0.6%)	18	39
East Asia	26 (0.3%)	5	298 (0.4%)	16	31
Eastern Europe	8 (0.1%)	3	8 (0.0%)	2	67
Eastern sub-Saharan Africa	44 (0.5%)	14	1,459 (21%)	28	50
High-income Pacific Asia	78 (0.9%)	6	221 (0.3%)	14	33
High-income North America	7,238 (79.4%)	14	24,552 (34.7%)	22	64
North Africa and Middle East	51 (0.6%)	14	542 (0.8%)	20	30
Oceania	0 (0.0%)	0	284 (0.4%)	6	N/A
South Asia	426 (4.7%)	9	115 (0.2%)	19	33
Southeast Asia	31 (0.3%)	13	1,008 (1.4%)	41	26
Southern Latin America	0 (0.0%)	0	554 (0.8%)	13	N/A
Southern sub-Saharan Africa	76 (0.8%)	11	2,551 (3.6%)	18	61
Tropical Latin America	8 (0.1%)	5	1,529 (2.2%)	19	42
Western Europe	219 (2.4%)	12	6,905 (9.8%)	14	73
Western sub-Saharan Africa	19 (0.2%)	10	2,007 (2.8%)	24	36
**Average Standard Deviation**	**434 ± 1,568**	**8 ± 7**	**3,367 ± 6,481**	**18 ± 11**	**48 ± 20**

^a^ Percentage of snake species matched between citizen-generated and scientist-generated sources of observational data

**Abbreviations:** GBD, global burden of disease; MIVS, medically important venomous snakes; N/A, not applicable.

**Table 3:** Most commonly observed MIVS species in citizen-generated and scientist-generated data sources per GBD region.

GBD regions	Citizen-generated observations *Species* (no of observations; %)	Scientist-generated observations *Species* (no of observations; %)
Andean Latin America	*Bothrops asper* (*n* = 13; 29.6%)	*B*. *atrox* (*n* = 310; 51.2%)
Australasia	*Notechis scutatus* (*n* = 26; 30.6%)	*Pseudonaja textilis* (*n* = 4,445; 24.8%)
Caribbean	*B*. *asper* (*n* = 6; 75.0%)	*B*. *atrox* (*n* = 64; 32.0%)
Central Asia	*Macrovipera lebetina* (*n* = 8; 32.0%)/ *Vipera eriwanensis* (*n* = 8; 32.0%)	*Gloydius halys* (*n* = 16; 32.0%)
Central Europe	*V*. *ammodytes* (*n* = 21; 87.5%)	*V*. *ammodytes* (*n* = 13; 40.6%)/ *V*. *berus* (*n* = 13; 40.6%)
Central Latin America	*C*. *atrox* (*n* = 114; 16.5%)	*C*. *atrox* (*n* = 1,346; 14.3%)
Central sub-Saharan Africa	*Atheris squamigera* (*n* = 3; 30.0%)	*Naja melanoleuca* (*n* = 111; 26.3%)
East Asia	*Bungarus multicinctus* (*n* = 10; 38.5%)	*Rhabdophis tigrinus* (*n* = 112; 37.6%)
Eastern Europe	*V*. *berus* (*n* = 5; 62.5%)	*G*. *blomhoffii* (*n* = 5; 62.5%)
East sub-Saharan Africa	*Bitis arietans* (*n* = 9; 20.5%)	*B*. *arietans* (*n* = 262; 18.0%)
High-income Asia Pacific	*R*. *tigrinus* (*n* = 54; 69.2%)	*R*. *tigrinus* (*n* = 138; 62.4%)
High-income North America	*C*. *atrox* (*n* = 1,808; 25.0%)	*C*. *viridis* (*n* = 6,994; 28.5%)
North Africa and Middle East	*Echis omanensis* (*n* = 15; 29.41%)	*Cerastes cerastes* (*n* = 156; 28.8%)
Oceania	*-*	*Acanthophis antarcticus* (*n* = 92; 32.4%)
South Asia	*Ophiophagus hannah* (*n* = 387; 90.9%)	*E*. *carinatus* (*n* = 30; 26.1%)
Southeast Asia	*Tropidolaemus subannulatus* (*n* = 10; 32.3%)	*Protobothrops mucrosquamatus* (*n* = 102; 10.1%)
Southern Latin America	*-*	*C*. *durissus* (*n* = 302; 54.5%)
Southern sub-Saharan Africa	*B*. *arietans* (*n* = 31; 40.8%)	*B*. *arietans* (*n* = 444; 17.4%)
Tropical Latin America	*C*. *durissus* (*n* = 3; 37.5%)	*B*. *jararaca* (*n* = 459; 30.0%)
Western Europe	*V*. *aspis* (*n* = 95; 43.4%)	*V*. *aspis* (*n* = 3,818; 55.3%)
Western sub-Saharan Africa	*N*. *melanoleuca* (*n* = 4; 21.1%)	*N*. *melanoleuca* (*n* = 313; 15.6%)

**Abbreviations:** GBD, Global burden of disease; MIVS, medically important venomous snakes.

Both the number of citizen-generated (*p* = 0.037, r = −0.48, *n* = 19) and scientist-generated observations (*p* = 0.019, r = −0.50, *n* = 21) were negatively correlated with snakebite mortality rates across GBD regions (*n* = 21). The points-in-polygon analysis revealed that 98.7% (*n* = 1,774), 91.6% (*n* = 1,295), 99.5% (*n* = 1,169), and 99.7% (*n* = 1,146) of citizen-generated observations gathered from iNaturalist for *C*. *atrox*, *C*. *oreganus*, *A*. *contortrix*, and *A*. *piscivorus* respectively were within their known species geographical range for the US in the period 1990–2017. Their geographical distributions in the US are illustrated in the supporting information (S1 Fig).

## Participatory approaches and open data: Opportunities and challenges

We gathered an unprecedented set of 79,810 georeferenced MIVS observations from all GBD regions of the world and built the first global observational map of MIVS that highlights the contribution of volunteer citizens, mobile technologies, and open participatory platforms for a rapid collection and public sharing of snake data. We found a severe geographical bias, with high-income North America concentrating most MIVS observations but very low snakebite mortality (<10 deaths/year), while GBD regions with the highest snakebite mortality (>1,000 deaths/year) represented only 5% of observations [12]. Our study provides insights on the quality of citizen-generated observations and crowdsourced identifications, opening innovative opportunities for contributions by citizens and collaborations with experts to study snakes in the context of the global snakebite crisis.

Snakes attract a global community of citizens and snake enthusiasts instantly and continuously sharing experiences and observations on open platforms such as iNaturalist. These observations are collected from wild and/or urban habitats worldwide, offering large volumes of data to complement the efforts of experts, whose data collection is bound to specific regions, species, and/or periods of the year depending on their research objectives [8]. We found that citizen-generated and scientist-generated observations correlated for the US and that 64% of them identified the same MIVS species. Besides, 97.3% of citizen-generated observations gathered from iNaturalist for *C*. *atrox*, *C*. *oreganus*, *A*. *contortrix*, and *A*. *piscivorus* were within their known species geographical range. More widely, the most frequently observed species and genera coincided between the two types of observations for 10 GBD regions. This highlights the quantitative and qualitative value of citizen-generated observations. Although we could potentially expect a reporting bias with a focus on large photogenic species by citizen-generated observations, these observations also include smaller and more cryptic MIVS species (e.g., *Echis omanensis*, *Atheris squamigera*, etc.), which seems to indicate that our iNaturalist community is interested in snake biodiversity in its widest sense. For instance, on September 1, 2017, the top five users with the most species on our iNaturalist project had each on average contributed sightings of 20 different MIVS species.

Although citizen participation has been used in the field of emerging zoonoses [7], its potential remains largely unexploited for snake ecology and its public health implications. The growing mobile technology markets in emerging and developing countries [13] open new opportunities for innovative and dynamic data collection and analysis based on citizen participation to improve our understanding of snake ecology and snakebite eco-epidemiology, particularly with rapid anthropogenic changes (e.g., climate change and urbanization [5, 8]).

The strong geographical bias toward North America in our citizen-generated observations matches previously observed gaps in snakebite research [14]. This could be explained in part by the American origin and development of the CS movement in the 1990s [15, 16]. The democratizing and transparent culture of CS could clash with many sociocultural and political systems [7] and limit its penetration in certain regions of the world. iNaturalist was launched in California in 2008, and despite its massive and global growth for some taxa (e.g., birds), it still remains undiscovered by many herpetology enthusiasts in emerging and developing countries. On the other hand, the gaps in scientist-generated observations from GBIF and VertNet are also severe and could be due to lack of funding, political and logistical challenges, and/or the risks of working with dangerous snakes in the poorest areas of the world. However, scientist-generated observations from GBIF and VertNet suffer less from the geographical bias toward high-income North America compared to citizen-generated observations. This can be explained by the lower temporal bias in scientist-generated observations from GBIF and VertNet due to their much longer time span (the period 1700–2016) [17] and to the great diversity of international partners around the world. Additionally, it is important to evaluate this global distribution map of MIVS species critically, as it might be biased by the unavailability of web-based technologies for CS activities in the poorest regions of the world or by field stations for research institutions (leading to false conclusions on the presence and abundance of certain MIVS species and subsequent risk of snakebite for local populations). Another similar challenge for open-access biodiversity platforms such as GBIF and VertNet is the Wallacean shortfall [17]. Due to incomplete records of compiled observations from different surveys or improper coverage of certain species along spatiotemporal and environmental dimensions [17], the geographical distribution of species is sometimes flawed by many gaps, resulting in a poor understanding of their ranges [18].

Despite these challenges, CS and open biodiversity platforms could offer a valuable source of information and expertise to be further exploited innovatively to better understand MIVS distribution in a more dynamic and local fashion. For example, the creation of a specific platform with targeted objectives dedicated to collect new contributions on venomous snake observations from enthusiasts could help in understanding their distribution more extensively. An interesting CS model is the one developed by the Cornell Lab of Ornithology, whose CS projects gather millions of species observations annually [19]. Such a CS project would require not only an interdisciplinary team of educators, evaluators, scientists, and technologists to monitor, promote, and safeguard the integrity of the project [19] but also ethicists to ensure that participants are not exposed to unintentional health risks and that their personal data are protected. Moreover, previously field-tested protocols and formally designed data collection forms would be required for data homogeneity and completeness of snake observations, minimization of bias during data collection, and an easier analysis of collected data from different regions of the world [19]. Additionally, CS projects could create links between volunteer citizens and experts and between society and universities, which would in turn ensure data quality of observations made by volunteer citizens and broaden the geographic scope of university research projects on venomous snake species. Furthermore, this CS project could gather data on snakebite-induced morbidity and loss of productivity in a relatively easy and cost-effective way through information gathering from afflicted communities by users. This would otherwise prove to be difficult and expensive since the only reliable method would be epidemiological community-based studies [20]. It is also worth noting that our iNaturalist project received quality contributions from volunteer herpetologists, university professors, and professional wildlife photographers. Additionally, the iNaturalist platform anticipated the time delay problem with the crowdsourced identification of species by its community and is now integrating machine learning techniques, such as computer vision, to provide automated higher-quality taxonomic identification of species uploaded on the platform [21].

CS initiatives could be powerful tools for educating and raising awareness on snakebite [7, 8] but would require very careful consideration to minimize risks and sociocultural rejection. Public participation could be particularly encouraged in regions where data is severely lacking. This could be done by creating partnerships with local universities, student associations interested in conservation biology or herpetology (e.g., zoology groups), local conservation groups, and snake catchers or rescuers. It could also be done through public service announcements, local workshops [19], and educational campaigns in communities. One successful example is the ongoing “Big 4 Mapping” project, which was launched in 2017 to map the distribution of four MIVS species (*Daboia russelii*, *Naja naja*, *Bungarus caeruleus*, and *E*. *carinatus*) responsible for over 90% of snakebite-induced deaths in India by employing a network of mainly snake catchers and/or rescuers [22]. In just a few months, it mapped over 1,600 snake observations in India [22]. This distribution map could then serve as a guide for effective antivenom distribution in snakebite hyperendemic areas [22]. Additionally, community leaders, traditional healers, village headmen, and local public health institutions could also be involved directly, such as in the recruitment of possible participants. The CS project would also benefit from adapting to the literacy level of its users, particularly when poor and vulnerable communities have low literacy rates. For example, 38% of the African population is nonliterate [23], and the use of standard data-collecting forms on smartphones might prove to be challenging in this situation. One successful CS project, Cybertracker (www.cybertracker.org), allows literate and nonliterate users to collect complex and rich geolocalized data on species distribution and behavior [24] and even provides recommendations and safety measures when tracking dangerous animals (including venomous snake species [25]). One interesting result of its application was its role as an early surveillance system for Ebola outbreaks in wildlife from 2001–2003, alerting authorities before these outbreaks constituted a public health threat in the Republic of Congo and Gabon [24]. Similar approaches could potentially be considered in the context of snake detection and identification as well as snakebite prevention and management. Participants could also be trained in safety and first aid techniques against snakebite, opening the door to collaboration with academia and health institutions (e.g., hospitals). Training programs with certified courses have been shown to increase adherence in previous CS projects [19].

In the context of snakebite management, taking a photo of the biting snake could be critical for a subsequent correct administration of antivenom or other lifesaving care. This could reduce the risks of new bites. In many regions of the world, the victim or bystanders usually try to kill and carry the snake to the clinician for identification. However, clinicians are not usually trained in herpetology, and their capacity to identify the snake is generally very limited. Urgent crowdsourced expertise with potentially massive global contributions (e.g., via the iNaturalist community or others) and/or machine learning systems based on computer vision offer potentially interesting decision support tools for clinicians to identify snakes via photos. Observations and associated photos such as those gathered in this study are valuable material to train computers in snake identification. Although promising molecular diagnostic tools could make a substantial contribution in reducing snakebite-induced morbidity and mortality in afflicted regions, they are still not fit to be used as point-of-care testing devices [26]. A pilot study done by Sharma et al. [26] has shown that in resource-limited settings, the time delay for seeking help by the bitten person or the use of inappropriate first-aid techniques (e.g., tourniquet) reduce the sensitivity of these molecular diagnostic techniques. Digital innovations such as crowdsourcing and machine learning (e.g., computer vision) could be complementary to molecular diagnostic tools. In some cases, these could be the only solution for resource-limited health centers, which do not have the financial and technical resources to implement and sustain some of these tools. Nonetheless, these approaches would only effectively reduce the snakebite burden in afflicted regions if the current antivenom crisis is addressed. For example, pharmaceutical companies have no market incentive for antivenom production, and Africa is running out of one of the most effective antivenoms against vipers and mambas [2, 27]. The current antivenom shortage and inadequate distribution of antivenom in different parts of the world is costing many lives. Urgent solutions with possible innovative incentives and mechanisms for accelerated research and implementation are needed.

The renewed momentum at the international level, through reintroduction of snakebite in WHO’s NTD list at WHA 70 in Geneva, should help raise funds to tackle snakebite in most afflicted regions and promote research in the field of antivenomics by designing improved polyspecific antivenoms [28] effective against the venoms of African and Asian MIVS. These could partially help in tackling the current antivenom crisis.

Alternative and complementary tools to antivenom should also be explored using scientific research and innovation (e.g., digital technologies) to tackle snakebite at different levels such as prevention and in different geographical contexts. Snakebite is not only a humanitarian crisis but also a data crisis. This study illustrates for the first time the potential of participatory approaches and citizen-generated data in this context. This innovative social and digital approach could contribute to data collection on snake ecology (e.g., geographical distribution of snakes) and subsequent snakebite epidemiology (e.g., hotspots of snakebite risk). For example, finer geographical maps of MIVS distribution could help to make public health interventions more specific by distributing the current limited supplies of antivenom where most needed. Careful consideration should also be given to the anticipation and prevention of potential harm for participants in this approach through a comprehensive strategy involving key stakeholders (e.g., members of afflicted communities, academia, public health entities, etc.) and adapted to the local socio-cultural context and health systems.

## Supporting information

S1 Text Detailed version of methodology. (DOCX)

S1 Fig Geographical distribution of *C*. *atrox*, *C*. *oreganus*, *A*. *contortrix*, and *A*. *piscivorus* citizen-generated observations (1990–2017) over known geographic range in the US. S1 Fig shows the geographical distribution of citizen-generated observations of *C*. *atrox*, *C*. *oreganus*, *A*. *contortrix*, and *A*. *piscivorus* species gathered from iNaturalist over their known geographical range. The time frame of observations is from 1990–2017. Source: Made with Natural Earth. Free vector and raster map data at www.naturalearthdata.com. The map (countries cultural theme version 3.1.0) is adapted and projected in the World Robinson coordinate reference system on QGIS 2.18.2. Species range data source: A. NatureServe and IUCN 2007. *C*. *atrox*. In: IUCN 2017. IUCN Red List of Threatened Species. Version 2017.2. http://www.iucnredlist.org. Downloaded on December 2, 2017; B. NatureServe and IUCN 2007. *C*. *oreganus*. In: IUCN 2017. IUCN Red List of Threatened Species. Version 2017.2. http://www.iucnredlist.org. Downloaded on December 2, 2017; C. NatureServe and IUCN 2007. *A*. *contortrix*. In: IUCN 2017. IUCN Red List of Threatened Species. Version 2017.2. http://www.iucnredlist.org. Downloaded on December 2, 2017 and D. NatureServe and IUCN 2007. *A*. *piscivorus*. In: IUCN 2017. IUCN Red List of Threatened Species. Version 2017.2. http://www.iucnredlist.org. Downloaded on December 2, 2017. **Abbreviations:** IUCN, International Union for Conservation of Nature.
(TIFF)

S1 Dataset GBIF Datasets used in study. (DOCX)

S2 Dataset VertNet records used in study. (XLSX)

S3 Dataset Citizen-generated observations dataset. (XLS)

S4 Dataset Scientist-generated observations dataset. (XLSX)
