# Supplementary material for: Participatory approaches and open data on venomous snakes: A neglected opportunity in the global snakebite crisis?
Source: PLoS Negl Trop Dis. 2018 Mar 8;12(3):e0006162. doi: 10.1371/journal.pntd.0006162 (PMC5843214; doi:10.1371/journal.pntd.0006162)
Supplement: S1 Text — (DOCX) [file pntd.0006162.s001.docx]

# **S1 Detailed version of methodology**

We created a project “*Medically Important Venomous Snakes*” on the *iNaturalist* platform on the 4^th^ of February 2017 and gathered existing MIVS [1] geolocalized observations from *iNaturalist* user accounts. *iNaturalist* (http://www.inaturalist.org) is one of the most important open-access platforms on biodiversity based on citizen participation and crowdsourced taxonomic identifications (over 3.9 million observations and 10,000 projects) and has been actively used in ecological research [2]. *iNaturalist* is a worldwide heterogeneous community of scientists, public volunteers and naturalists which assist and help each other in producing high quality CS and conservation data [3]. The MIVS observations were manually transferred from *iNaturalist* user accounts to our project from the 4^th^ to the 15^th^ of February 2017, with no prerequisite time frame for the gathered observations. We had no intention to set up a new portal for collecting MIVS observational data but rather to gather existing MIVS observations through the creation of a new project on the *iNaturalist* platform. This new *iNaturalist* project can be viewed as a sub-portal within the *iNaturalist* platform, benefiting directly from *iNaturalist’s* community of data contributors*. Only “research grade”* were considered to ensure quality of identifications. These are defined by *iNaturalist* as observations having 2/3 of *iNaturalist* community agreement on their taxonomic identification. Additionally, the *iNaturalist* community should also agree that observations provide evidence on the accuracy of the location, the wild nature of the organism, and that the submitted photos provide recent evidence of the organism (e.g. fossils are excluded). Additional geolocalized observations were collected from five CS projects available on *GBIF*. After removal of redundant observations between *iNaturalist* and *GBIF*, all observations were merged into a single dataset (*citizen-generated* observations). These observations are generated by a potentially heterogeneous community of volunteer citizens with or without scientific background and with possible different motivations. A second dataset consisting of traditional sources of geolocalized MIVS observations (*scientist-generated* observations) was built from *GBIF* and *VertNet* on the 26^th^ February 2017. These observations are generated by scientists in the field as part of their research projects. *GBIF* and *VertNet*, with over 715 and 50 million observations respectively, are massive open-access biodiversity platforms that gather global information on animals, observed mainly by professionals from Museums and Universities [4]. Data quality for *GBIF* and *VertNet* is ensured through standard protocols (Darwin core) and further data quality checks performed by these respective platforms for their published datasets [4, 5]. All the observations considered and coming from these three sources of data (*iNaturalist*, *GBIF* and *VertNet*) were identified at the level of species (genus and specific epithet). Observations lacking GPS coordinates were excluded from the study. Although we cannot exclude some possible inaccuracies for the locations of snake observations coming from *iNaturalist, GBIF* and *VertNet*, which we were unable to account for in our datasets, the large sample sizes of the *citizen-generated* and *scientist-generated* observations datasets reduce possible impact on statistical analysis and overall distribution patterns [6].

## **Data analysis**

A total of 9,113 *citizen-generated* (8,520 from *iNaturalis*t and 593 from CS projects from *GBIF*) and 70,697 *scientist-generated* observations were statistically and spatially analysed using STATA 14 and QGIS 2.18.2. Observations were mapped on public domain vector map data made with *Natural Earth* (www.naturalearthdata.com) using QGIS. Descriptive statistics for Global Burden of Disease (GBD) regions were obtained for both type of observations. GBD regions were chosen to test for association between collected MIVS observational data from *iNaturalist*, *GBIF* and *VertNet*, and GBD estimates of snakebite-induced mortality rates from Kasturiratne et al. (2008) [7]. GBD regions are 21 different geographical regions used to group 277 countries around the world, based on similar epidemiological patterns, for the 2005 Global Burden Project of the World Bank [7]. Estimates of envenoming and mortality were obtained from Kasturiratne et al. (2008) [7] to calculate the mortality rate (per 100,000 envenomings) per GBD region and a linear regression analysis was performed between the log transformed mortality rate and the number of observations. However, we cannot guarantee the robustness of the data obtained from Kasturiratne et al. (2008) [7] since the lack of the required heterogeneity in snakebite incidence estimates within and between countries and their generalization across neighbouring regions have led to wide annual snakebite envenoming and mortality rates in their study [8]. To infer the spatial congruence between both types of observations, we performed a linear regression analysis between the log-transformed numbers of *citizen-generated* and *scientist-generated* observations. This test was performed for observations spanning the period 1990-2017 and only for the USA, where the highest number of observations are found. We tested the following grid resolutions over the USA to quantify log-transformed numbers of observations: 40X40km^2^; 60X60km^2^; 80X80km^2^; 100X100km^2^; 120X120km^2^; 160X160km^2^; 200X200km^2^; 300X300km^2^. Moreover, distribution patterns of the four most frequently observed MIVS (*Agkistrodon contortrix*, *Agkistrodon piscivorus*, *Crotalus atrox* and *Crotalus oreganus*), in our *citizen-generated* observations, were compared to their respective species range maps in the USA. The time frame of observations was 1990-2017 and the species range maps were provided by the International Union for Conservation of Nature (IUCN). A points-in-polygon analysis was carried out for each species to determine the percentage of observations falling within range.

# **References**

1. WHO Guidelines for the Production, Control and Regulation of Snake Antivenom Immunoglobulins [Internet]. 1st ed. Geneva: World Health Organization; 2010 [cited 10 March 2017]. Available from: http://www.who.int/bloodproducts/snake_antivenoms/SnakeAntivenomGuideline.pdf
2. White RL, Sutton AE, Salguero-Gomez R, Bray TC, Campbell H, Cieraad E, et al. The next generation of action ecology: novel approaches towards global ecological research. Ecosphere. 2015 Aug 1;6(8):1-16
3. iNaturalist Network [Internet]. iNaturalist.org. [cited 2017 Sep 7]. Available from: https://www.inaturalist.org/pages/network
4. Constable H, Guralnick R, Wieczorek J, Spencer C, Peterson AT, Committee TVS. VertNet: A New Model for Biodiversity Data Sharing. PLOS Biology. 2010 Feb 16;8(2):e1000309.
5. Global Biodiversity Information Facility. Data quality requirements [Internet]. GBIF.ORG. 2016 [cited 2017 Aug 7]. Available from: http://www.gbif.org/publishing-data/quality
6. Bonney R, Cooper CB, Dickinson J, Kelling S, Phillips T, Rosenberg KV, et al. Citizen Science: A Developing Tool for Expanding Science Knowledge and Scientific Literacy. BioScience. 2009 Dec 1;59(11):977–84.
7. Kasturiratne A, Wickremasinghe AR, de Silva N, Gunawardena NK, Pathmeswaran A, Premaratna R, et al. The global burden of snakebite: a literature analysis and modelling based on regional estimates of envenoming and deaths. PLoS Med. 2008 Nov 4;5(11):e218.
8. Warrell DA. Snake bite. Lancet. 2010 Jan 2;375(9708):77–88.
