# Supplementary figures and images for: Participatory approaches and open data on venomous snakes: A neglected opportunity in the global snakebite crisis?
Source: PLoS Negl Trop Dis. 2018 Mar 8;12(3):e0006162. doi: 10.1371/journal.pntd.0006162 (PMC5843214; doi:10.1371/journal.pntd.0006162)

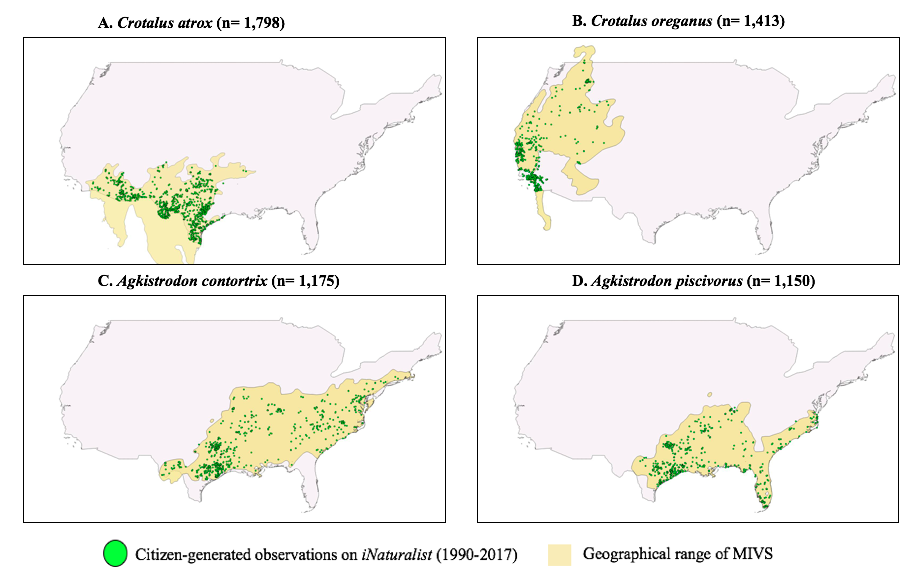

Supplement: S1 Fig — shows the geographical distribution of citizen-generated observations of C. atrox, C. oreganus, A. contortrix, and A. piscivorus species gathered from iNaturalist over their known geographical range. The time frame of observations is from 1990–2017. Source: Made with Natural Earth. Free vector and raster map data at www.naturalearthdata.com. The map (countries cultural theme version 3.1.0) is adapted and projected in the World Robinson coordinate reference system on QGIS 2.18.2. Species range data source: A. NatureServe and IUCN 2007. C. atrox. In: IUCN 2017. IUCN Red List of Threatened Species. Version 2017.2. http://www.iucnredlist.org. Downloaded on December 2, 2017; B. NatureServe and IUCN 2007. C. oreganus. In: IUCN 2017. IUCN Red List of Threatened Species. Version 2017.2. http://www.iucnredlist.org. Downloaded on December 2, 2017; C. NatureServe and IUCN 2007. A. contortrix. In: IUCN 2017. IUCN Red List of Threatened Species. Version 2017.2. http://www.iucnredlist.org. Downloaded on December 2, 2017 and D. NatureServe and IUCN 2007. A. piscivorus. In: IUCN 2017. IUCN Red List of Threatened Species. Version 2017.2. http://www.iucnredlist.org. Downloaded on December 2, 2017. Abbreviations: IUCN, International Union for Conservation of Nature. (TIFF) [file pntd.0006162.s002.tiff]
