# Supplementary material for: Participatory approaches and open data on venomous snakes: A neglected opportunity in the global snakebite crisis?
Source: PLoS Negl Trop Dis. 2018 Mar 8;12(3):e0006162. doi: 10.1371/journal.pntd.0006162 (PMC5843214; doi:10.1371/journal.pntd.0006162)
Supplement: S1 Dataset — (DOCX) [file pntd.0006162.s003.docx]

# GBIF Datasets used in study

GBIF.org (26th February 2017) GBIF Occurrence Download <http://doi.org/10.15468/dl.bbbe3f>

GBIF.org (26th February 2017) GBIF Occurrence Download <http://doi.org/10.15468/dl.0wuewa>

  GBIF.org (26th February 2017) GBIF Occurrence Download <http://doi.org/10.15468/dl.vd3pre>

GBIF.org (26th February 2017) GBIF Occurrence Download <http://doi.org/10.15468/dl.z0kj1w>

GBIF.org (26th February 2017) GBIF Occurrence Download <http://doi.org/10.15468/dl.iv5srj>

GBIF.org (26th February 2017) GBIF Occurrence Download <http://doi.org/10.15468/dl.encknb>

GBIF.org (26th February 2017) GBIF Occurrence Download <http://doi.org/10.15468/dl.o4hlw5>

GBIF.org (26th February 2017) GBIF Occurrence Download <http://doi.org/10.15468/dl.bklyhf>

GBIF.org (26th February 2017) GBIF Occurrence Download <http://doi.org/10.15468/dl.rjtybl>

GBIF.org (26th February 2017) GBIF Occurrence Download <http://doi.org/10.15468/dl.u7zb55>

  GBIF.org (26th February 2017) GBIF Occurrence Download <http://doi.org/10.15468/dl.c6axms>

  GBIF.org (26th February 2017) GBIF Occurrence Download <http://doi.org/10.15468/dl.xhbjm4>

GBIF.org (26th February 2017) GBIF Occurrence Download <http://doi.org/10.15468/dl.oxalpg>

GBIF.org (26th February 2017) GBIF Occurrence Download <http://doi.org/10.15468/dl.fbtm1i>

GBIF.org (26th February 2017) GBIF Occurrence Download <http://doi.org/10.15468/dl.kvpohg>

GBIF.org (26th February 2017) GBIF Occurrence Download <http://doi.org/10.15468/dl.qflj8j>

GBIF.org (26th February 2017) GBIF Occurrence Download <http://doi.org/10.15468/dl.yu1bye>

GBIF.org (26th February 2017) GBIF Occurrence Download <http://doi.org/10.15468/dl.9ipshw>

GBIF.org (26th February 2017) GBIF Occurrence Download <http://doi.org/10.15468/dl.rkjykm>

GBIF.org (26th February 2017) GBIF Occurrence Download <http://doi.org/10.15468/dl.cherhv>

  GBIF.org (26th February 2017) GBIF Occurrence Download <http://doi.org/10.15468/dl.2b9nqy>

  GBIF.org (26th February 2017) GBIF Occurrence Download <http://doi.org/10.15468/dl.kzcaly>

GBIF.org (26th February 2017) GBIF Occurrence Download <http://doi.org/10.15468/dl.cxffgt>

  GBIF.org (26th February 2017) GBIF Occurrence Download <http://doi.org/10.15468/dl.vganms>

  GBIF.org (26th February 2017) GBIF Occurrence Download <http://doi.org/10.15468/dl.53ndtt>

GBIF.org (26th February 2017) GBIF Occurrence Download <http://doi.org/10.15468/dl.8ptbmt>

GBIF.org (26th February 2017) GBIF Occurrence Download <http://doi.org/10.15468/dl.24okca>

  GBIF.org (26th February 2017) GBIF Occurrence Download <http://doi.org/10.15468/dl.9seona>

GBIF.org (26th February 2017) GBIF Occurrence Download <http://doi.org/10.15468/dl.ueikbw>

  GBIF.org (26th February 2017) GBIF Occurrence Download <http://doi.org/10.15468/dl.3t4gen>

GBIF.org (26th February 2017) GBIF Occurrence Download <http://doi.org/10.15468/dl.t8axy6>

  GBIF.org (26th February 2017) GBIF Occurrence Download <http://doi.org/10.15468/dl.sjbziv>

GBIF.org (26th February 2017) GBIF Occurrence Download <http://doi.org/10.15468/dl.pfoxtb>

GBIF.org (26th February 2017) GBIF Occurrence Download <http://doi.org/10.15468/dl.xrag7k>

GBIF.org (26th February 2017) GBIF Occurrence Download <http://doi.org/10.15468/dl.zux8ab>

  GBIF.org (26th February 2017) GBIF Occurrence Download <http://doi.org/10.15468/dl.gera7f>

  GBIF.org (26th February 2017) GBIF Occurrence Download <http://doi.org/10.15468/dl.ubcg4e>

GBIF.org (26th February 2017) GBIF Occurrence Download <http://doi.org/10.15468/dl.seueqd>

GBIF.org (26th February 2017) GBIF Occurrence Download <http://doi.org/10.15468/dl.kqetby>

  GBIF.org (26th February 2017) GBIF Occurrence Download <http://doi.org/10.15468/dl.0w4ir2>

  GBIF.org (26th February 2017) GBIF Occurrence Download <http://doi.org/10.15468/dl.wtk00s>

GBIF.org (26th February 2017) GBIF Occurrence Download <http://doi.org/10.15468/dl.f9pasy>

  GBIF.org (26th February 2017) GBIF Occurrence Download <http://doi.org/10.15468/dl.b14vda>

GBIF.org (26th February 2017) GBIF Occurrence Download <http://doi.org/10.15468/dl.am5fkz>

  GBIF.org (26th February 2017) GBIF Occurrence Download <http://doi.org/10.15468/dl.4xq9ya>

  GBIF.org (26th February 2017) GBIF Occurrence Download <http://doi.org/10.15468/dl.ghabqu>

GBIF.org (26th February 2017) GBIF Occurrence Download <http://doi.org/10.15468/dl.ttvqdc>

  GBIF.org (26th February 2017) GBIF Occurrence Download <http://doi.org/10.15468/dl.5p2epd>

GBIF.org (26th February 2017) GBIF Occurrence Download <http://doi.org/10.15468/dl.1ekm7d>

  GBIF.org (26th February 2017) GBIF Occurrence Download <http://doi.org/10.15468/dl.j1ge98>

  GBIF.org (26th February 2017) GBIF Occurrence Download <http://doi.org/10.15468/dl.c6mfec>

  GBIF.org (26th February 2017) GBIF Occurrence Download <http://doi.org/10.15468/dl.wanuk0>

GBIF.org (26th February 2017) GBIF Occurrence Download <http://doi.org/10.15468/dl.knejs5>

  GBIF.org (26th February 2017) GBIF Occurrence Download <http://doi.org/10.15468/dl.nohjnt>

  GBIF.org (26th February 2017) GBIF Occurrence Download <http://doi.org/10.15468/dl.lmvglw>

  GBIF.org (26th February 2017) GBIF Occurrence Download <http://doi.org/10.15468/dl.nkrlzh>

  GBIF.org (26th February 2017) GBIF Occurrence Download <http://doi.org/10.15468/dl.farvzu>

  GBIF.org (26th February 2017) GBIF Occurrence Download <http://doi.org/10.15468/dl.qowoar>

GBIF.org (26th February 2017) GBIF Occurrence Download <http://doi.org/10.15468/dl.mhxgfn>

  GBIF.org (26th February 2017) GBIF Occurrence Download <http://doi.org/10.15468/dl.3zggch>

  GBIF.org (26th February 2017) GBIF Occurrence Download <http://doi.org/10.15468/dl.ah5cqg>

GBIF.org (26th February 2017) GBIF Occurrence Download <http://doi.org/10.15468/dl.d9bylp>

GBIF.org (26th February 2017) GBIF Occurrence Download <http://doi.org/10.15468/dl.oxdsvi>

GBIF.org (26th February 2017) GBIF Occurrence Download <http://doi.org/10.15468/dl.pa5mf7>

GBIF.org (26th February 2017) GBIF Occurrence Download <http://doi.org/10.15468/dl.qktstu>

  GBIF.org (26th February 2017) GBIF Occurrence Download <http://doi.org/10.15468/dl.ycjhf7>

  GBIF.org (26th February 2017) GBIF Occurrence Download <http://doi.org/10.15468/dl.75jdfa>

  GBIF.org (26th February 2017) GBIF Occurrence Download <http://doi.org/10.15468/dl.ygscjv>

GBIF.org (26th February 2017) GBIF Occurrence Download <http://doi.org/10.15468/dl.p2bynh>

GBIF.org (26th February 2017) GBIF Occurrence Download <http://doi.org/10.15468/dl.qzkijf>

  GBIF.org (26th February 2017) GBIF Occurrence Download <http://doi.org/10.15468/dl.0ghmyh>

GBIF.org (26th February 2017) GBIF Occurrence Download <http://doi.org/10.15468/dl.j9egsk>

GBIF.org (26th February 2017) GBIF Occurrence Download <http://doi.org/10.15468/dl.flpwcm>

  GBIF.org (26th February 2017) GBIF Occurrence Download <http://doi.org/10.15468/dl.4lq31c>

GBIF.org (26th February 2017) GBIF Occurrence Download <http://doi.org/10.15468/dl.gax23d>

   GBIF.org (26th February 2017) GBIF Occurrence Download <http://doi.org/10.15468/dl.z7ayxh>

GBIF.org (26th February 2017) GBIF Occurrence Download <http://doi.org/10.15468/dl.0us4hr>

GBIF.org (26th February 2017) GBIF Occurrence Download <http://doi.org/10.15468/dl.qpdcfe>

GBIF.org (26th February 2017) GBIF Occurrence Download <http://doi.org/10.15468/dl.xptnme>

GBIF.org (26th February 2017) GBIF Occurrence Download <http://doi.org/10.15468/dl.4oxhv2>

GBIF.org (26th February 2017) GBIF Occurrence Download <http://doi.org/10.15468/dl.cx9e1v>

GBIF.org (26th February 2017) GBIF Occurrence Download <http://doi.org/10.15468/dl.clvblh>
  GBIF.org (26th February 2017) GBIF Occurrence Download <http://doi.org/10.15468/dl.ozcqll>

GBIF.org (26th February 2017) GBIF Occurrence Download <http://doi.org/10.15468/dl.b6mudl>

GBIF.org (26th February 2017) GBIF Occurrence Download <http://doi.org/10.15468/dl.e8204p>

   GBIF.org (26th February 2017) GBIF Occurrence Download <http://doi.org/10.15468/dl.te43pr>

GBIF.org (26th February 2017) GBIF Occurrence Download <http://doi.org/10.15468/dl.3kamdk>

GBIF.org (26th February 2017) GBIF Occurrence Download <http://doi.org/10.15468/dl.5kh9xv>

GBIF.org (26th February 2017) GBIF Occurrence Download <http://doi.org/10.15468/dl.1ajgjr>

GBIF.org (26th February 2017) GBIF Occurrence Download <http://doi.org/10.15468/dl.gzvisy>

  GBIF.org (26th February 2017) GBIF Occurrence Download <http://doi.org/10.15468/dl.9w8jgn>

GBIF.org (26th February 2017) GBIF Occurrence Download <http://doi.org/10.15468/dl.dsy1zs>

GBIF.org (26th February 2017) GBIF Occurrence Download <http://doi.org/10.15468/dl.jbyfzb>

GBIF.org (26th February 2017) GBIF Occurrence Download <http://doi.org/10.15468/dl.rdolde>

  GBIF.org (26th February 2017) GBIF Occurrence Download <http://doi.org/10.15468/dl.tffqk9>

  GBIF.org (26th February 2017) GBIF Occurrence Download <http://doi.org/10.15468/dl.lh4wxa>

  GBIF.org (26th February 2017) GBIF Occurrence Download <http://doi.org/10.15468/dl.cznevt>

  GBIF.org (26th February 2017) GBIF Occurrence Download <http://doi.org/10.15468/dl.xotwth>

GBIF.org (26th February 2017) GBIF Occurrence Download <http://doi.org/10.15468/dl.d6a3do>

GBIF.org (26th February 2017) GBIF Occurrence Download <http://doi.org/10.15468/dl.hwhvtx>

GBIF.org (26th February 2017) GBIF Occurrence Download <http://doi.org/10.15468/dl.6iowr0>

GBIF.org (26th February 2017) GBIF Occurrence Download <http://doi.org/10.15468/dl.kyuq0n>

  GBIF.org (26th February 2017) GBIF Occurrence Download <http://doi.org/10.15468/dl.lwhkla>

GBIF.org (26th February 2017) GBIF Occurrence Download <http://doi.org/10.15468/dl.l0zpvf>

  GBIF.org (26th February 2017) GBIF Occurrence Download <http://doi.org/10.15468/dl.gapilf>

  GBIF.org (26th February 2017) GBIF Occurrence Download <http://doi.org/10.15468/dl.ogkup3>

  GBIF.org (26th February 2017) GBIF Occurrence Download <http://doi.org/10.15468/dl.hpo4lu>

GBIF.org (26th February 2017) GBIF Occurrence Download <http://doi.org/10.15468/dl.bntu36>

GBIF.org (26th February 2017) GBIF Occurrence Download <http://doi.org/10.15468/dl.nmeitw>

GBIF.org (26th February 2017) GBIF Occurrence Download <http://doi.org/10.15468/dl.2nb2wx>

  GBIF.org (26th February 2017) GBIF Occurrence Download <http://doi.org/10.15468/dl.i2f83w>

GBIF.org (26th February 2017) GBIF Occurrence Download <http://doi.org/10.15468/dl.p9awzv>
GBIF.org (26th February 2017) GBIF Occurrence Download <http://doi.org/10.15468/dl.jxbueb>

  GBIF.org (26th February 2017) GBIF Occurrence Download <http://doi.org/10.15468/dl.sim1l7>

GBIF.org (26th February 2017) GBIF Occurrence Download <http://doi.org/10.15468/dl.lwtnqk>

   GBIF.org (26th February 2017) GBIF Occurrence Download <http://doi.org/10.15468/dl.btkidw>

    GBIF.org (26th February 2017) GBIF Occurrence Download <http://doi.org/10.15468/dl.bhkb2r>

    GBIF.org (26th February 2017) GBIF Occurrence Download <http://doi.org/10.15468/dl.hr3nsu>

    GBIF.org (26th February 2017) GBIF Occurrence Download <http://doi.org/10.15468/dl.chjd4b>

    GBIF.org (26th February 2017) GBIF Occurrence Download <http://doi.org/10.15468/dl.wohcik>

    GBIF.org (26th February 2017) GBIF Occurrence Download <http://doi.org/10.15468/dl.4sxq7e>

    GBIF.org (26th February 2017) GBIF Occurrence Download <http://doi.org/10.15468/dl.xuwgvo>

    GBIF.org (26th February 2017) GBIF Occurrence Download <http://doi.org/10.15468/dl.w1oo9w>

    GBIF.org (26th February 2017) GBIF Occurrence Download <http://doi.org/10.15468/dl.9ggyy3>

    GBIF.org (26th February 2017) GBIF Occurrence Download <http://doi.org/10.15468/dl.c4r9dl>

    GBIF.org (26th February 2017) GBIF Occurrence Download <http://doi.org/10.15468/dl.buhvqu>

    GBIF.org (26th February 2017) GBIF Occurrence Download <http://doi.org/10.15468/dl.54gybs>

    GBIF.org (26th February 2017) GBIF Occurrence Download <http://doi.org/10.15468/dl.4crwbj>

    GBIF.org (26th February 2017) GBIF Occurrence Download <http://doi.org/10.15468/dl.dmw6or>

    GBIF.org (26th February 2017) GBIF Occurrence Download <http://doi.org/10.15468/dl.mwarsy>

    GBIF.org (26th February 2017) GBIF Occurrence Download <http://doi.org/10.15468/dl.cgesfy>

    GBIF.org (26th February 2017) GBIF Occurrence Download <http://doi.org/10.15468/dl.tmbc2k>

    GBIF.org (26th February 2017) GBIF Occurrence Download <http://doi.org/10.15468/dl.bpxqy8>

    GBIF.org (26th February 2017) GBIF Occurrence Download <http://doi.org/10.15468/dl.lsrtph>

    GBIF.org (26th February 2017) GBIF Occurrence Download <http://doi.org/10.15468/dl.ee8aqv>

    GBIF.org (26th February 2017) GBIF Occurrence Download <http://doi.org/10.15468/dl.gysdj6>

    GBIF.org (26th February 2017) GBIF Occurrence Download <http://doi.org/10.15468/dl.rmfywy>

    GBIF.org (26th February 2017) GBIF Occurrence Download <http://doi.org/10.15468/dl.7wrgny>

    GBIF.org (26th February 2017) GBIF Occurrence Download <http://doi.org/10.15468/dl.aetj1v>

    GBIF.org (26th February 2017) GBIF Occurrence Download <http://doi.org/10.15468/dl.8aj6ei>

    GBIF.org (26th February 2017) GBIF Occurrence Download <http://doi.org/10.15468/dl.8254d7>

    GBIF.org (26th February 2017) GBIF Occurrence Download <http://doi.org/10.15468/dl.yl2kek>

    GBIF.org (26th February 2017) GBIF Occurrence Download <http://doi.org/10.15468/dl.4xs651>

    GBIF.org (26th February 2017) GBIF Occurrence Download <http://doi.org/10.15468/dl.s9npy6>

    GBIF.org (26th February 2017) GBIF Occurrence Download <http://doi.org/10.15468/dl.cdy3y1>

    GBIF.org (26th February 2017) GBIF Occurrence Download <http://doi.org/10.15468/dl.b7xbdf>

    GBIF.org (26th February 2017) GBIF Occurrence Download <http://doi.org/10.15468/dl.zti5xz>

    GBIF.org (26th February 2017) GBIF Occurrence Download <http://doi.org/10.15468/dl.beuvih>

    GBIF.org (26th February 2017) GBIF Occurrence Download <http://doi.org/10.15468/dl.mt9imr>

    GBIF.org (26th February 2017) GBIF Occurrence Download <http://doi.org/10.15468/dl.wr2mek>

    GBIF.org (26th February 2017) GBIF Occurrence Download <http://doi.org/10.15468/dl.tjiemn>

    GBIF.org (26th February 2017) GBIF Occurrence Download <http://doi.org/10.15468/dl.ac0cna>

    GBIF.org (26th February 2017) GBIF Occurrence Download <http://doi.org/10.15468/dl.jfrgwu>

    GBIF.org (26th February 2017) GBIF Occurrence Download <http://doi.org/10.15468/dl.hy3arf>

    GBIF.org (26th February 2017) GBIF Occurrence Download <http://doi.org/10.15468/dl.dq11th>

    GBIF.org (26th February 2017) GBIF Occurrence Download <http://doi.org/10.15468/dl.4ncqzo>
